# High serum uric acid level is associated with greater handgrip strength in the aged population

**DOI:** 10.1186/s13075-019-1858-2

**Published:** 2019-03-12

**Authors:** Jennifer Lee, Yeon Sik Hong, Sung-Hwan Park, Kwi Young Kang

**Affiliations:** 1 Division of Rheumatology, Department of Internal Medicine, Seoul St. Mary’s Hospital, College of Medicine, The Catholic University of Korea, Seoul, South Korea; 2 Division of Rheumatology, Department of Internal Medicine, Incheon St. Mary’s Hospital, College of Medicine, The Catholic University of Korea, #56, Dongsu-Ro, Bupyung-Gu, Incheon, South Korea

**Keywords:** Uric acid, Muscle strength, Hand strength, Antioxidants

## Abstract

**Background:**

We aimed to investigate the association of serum UA level with muscle strength assessed by handgrip strength (HGS) in a large Korean adult population.

**Methods:**

Cross-sectional data were obtained from the seventh Korea National Health and Nutrition Examination Survey (KNHANES) 2016. The KNHANES 2016 study included 8150 subjects, of whom 4230 subjects were analyzed in this study. The association between serum UA level and HGS was investigated with adjustment for confounding factors.

**Results:**

Serum UA was divided into sex-specific tertiles After adjustment for potential confounding factors, HGS was significantly greater in the high serum UA group (the third tertile) than in the low UA group (the first tertile) in the elderly (age ≥ 60 years) population (coefficient *β* [95% confidence interval (CI)] = 1.017 [0.115–1.920]). When the elderly population was subdivided according to the presence of metabolic syndrome (metS), the impact of UA remained significant only in individuals with metS. In the aged population, high serum UA level reduced the risk for low HGS (OR, 95% CI = 0.69, 0.48–0.98, *p* = 0.041) only in male subjects.

**Conclusions:**

A population-based cross-sectional survey in Korea revealed that high serum UA level is associated with increased HGS in the aged population. The antioxidant property of UA may enhance muscle strength, especially in the elderly population.

**Electronic supplementary material:**

The online version of this article (10.1186/s13075-019-1858-2) contains supplementary material, which is available to authorized users.

## Introduction

Uric acid (UA) is produced during the metabolism of the DNA component purine [1]. Uricase, which converts uric acid into soluble allantoin, is lacking in primates, so serum UA level can be maintained relatively high in primates compared to non-primates [2]. UA is known to be important for maintaining high blood pressure for upright walking and is a powerful antioxidant [3, 4]. These functions of UA have led to the concept that uricase was abolished in the evolutionary process that rendered UA essential [5]. However, UA can also exert detrimental effects as a prooxidant that enhances oxidative stress [6] or a danger-associated molecular pattern (DAMP) that provokes systemic inflammation [7]. Likewise, high levels of UA have been implicated in gout [8], cardiovascular mortality [9, 10], and hypertension [11], while low levels of UA have been reported to increase the risk of Alzheimer’s disease [12] and fracture [13]. The known physiological and pathological roles of UA have been expanding [14].

Sarcopenia is defined by both low muscle mass and low muscle function [15]. Sarcopenia develops in the normal aging process, but also can develop secondary to medical conditions and is associated with gerontologic problems such as pain in osteoarthritis [16] and mortality [17]. Among various risk factors, oxidative stress on the muscle has been suggested to play a critical role in sarcopenia [18], raising the question of how UA (which has both pro- and antioxidant properties) affects sarcopenia.

The vast majority of studies on sarcopenia have been conducted in the elderly population, while studies on the younger population are rare. In the elderly population, most studies have reported the beneficial effects of UA on muscle strength [19–22]; nevertheless, the opposite results have also been reported [23]. Two recent studies that included younger subjects had inconsistent results [24, 25], likely due to differences in study design and age range of the subjects.

In Korea, a national survey system called the Korea National Health and Nutrition Examination Survey (KNHANES) has been performed since 1998. The KNHANES in 2016 included serum UA level for the first time, and muscle strength was measured based on handgrip strength (HGS), although muscle mass could not be evaluated. Using these data, we aimed to investigate the association of serum UA level with muscle strength after adjusting for various confounding factors in a large unbiased nationwide population.

## Methods

### Study participants

The KNHANES is a nationwide, population-based, cross-sectional health examination and survey that has been conducted every year since 1998 by the Division of Chronic Disease Surveillance of the Korea Centers for Disease Control and Prevention in the Ministry of Health and Welfare to monitor the general health and nutrition status of the non-institutionalized civilian population of South Korea (Ref: Korea Centers for Disease Control and Prevention. The Seventh Korea National Health and Nutrition Examination Survey (KNHANES VII) 2016. Seoul, Korea: Division of Chronic Disease Surveillance, Korea Centers for Disease Control and Prevention). Thus, every year, 8000–10,000 individuals from 4600 households are selected as representative Koreans. This selection is performed through a multi-stage clustered and stratified random sampling method based on national census data. The survey is composed of three individual surveys, namely, a health interview, a nutrition survey, and a health examination survey. The data are collected through household interviews and standardized physical examinations that are conducted at mobile examination centers. The KNHANES database is publicly available at the KNHANES website (http://knhanes.cdc.go.kr, available in Korean). The present study was based on the participants in the first (2016) year of the seventh KNHANES.

**Fig. 1:**
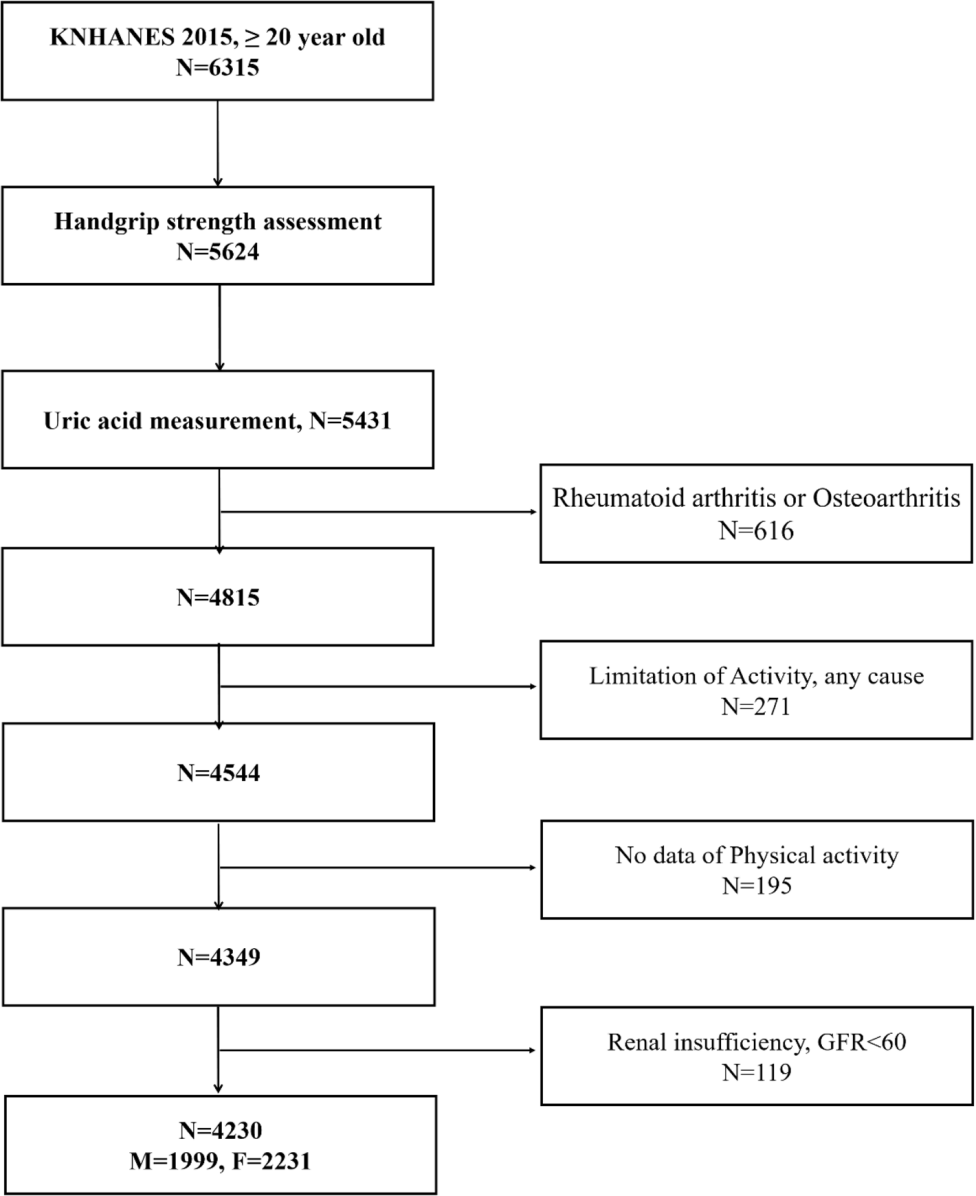
Selection of the study participants

The KNHANES VII-1 (2016) examination and health survey was completed by 8150 participants. The following individuals were included in this analysis: those 20 years and above (*N* = 6315) who were assessed for HGS (*N* = 5624). Of the 5624 subjects with HGS data, only those whose serum uric acid data were collected were included (*N* = 5431). The following subjects were excluded: subjects who were diagnosed with rheumatoid arthritis or osteoarthritis by a doctor (*N* = 616), subjects with limitation of physical activity (*N* = 271), subjects who did not respond to the physical activity survey (*N* = 195), and subjects with an estimated glomerular filtration rate (eGFR) less than 60 mL/min/1.73 m^2^ (*N* = 119). The remaining 4230 subjects (1999 men and 2231 women) were eligible for this study (Fig. 1).

Written informed consent was secured from all participants before the survey began. The study was approved by the ethics committee at Seoul Saint Mary’s Hospital (KC18ZESI0273).

### Measurement of HGS

HGS was measured with a digital grip strength dynamometer (TKK 5401 GRIPD; Takei, Japan), which measures forces between 5.0 and 100.0 kg and has an adjustable grip span. The minimum measurement unit is 0.1 kg. During the assessment, participants were asked to stand upright with their feet hip-width apart and to look forward with their elbow fully extended. The dynamometer was held by the testing hand in a neutral, comfortable position (not flexed or extended) with 90° flexion at the index finger. Participants were instructed to squeeze the grip continuously with full force for at least 3 s. They were asked not to swing the grip dynamometer during the test and not to hold their breath. The time between each trial was approximately 60 s. Each hand was tested three times, and the highest reading from the dominant hand was used as maximum grip strength, expressed in kilograms [26]. Low HGS was defined as < 26 kg for men and < 18 kg for women [27].

### Measurement of serum UA

Blood samples were drawn from the antecubital vein in the morning after an overnight fast. Serum UA level was measured by a colorimetric enzymatic method on a Hitachi 7600-210 automatic biochemical analyzer (Hitachi, Japan). UA levels were then divided into sex-specific tertiles based on their distribution: ≤ 5.2, 5.3–6.2, and ≥ 6.3 mg/dL in men and ≤ 3.9, 4.0–4.6, and ≥ 4.7 mg/dL in women. Hyperuricemia was defined as a serum UA level of ≥ 7.0 mg/dL for men and ≥ 6.0 mg/dL for women [28, 29].

### Study variables

Age, weight, height, smoking, alcohol drinking, household income, and physical activity were recorded. Weight and height were collected according to standardized procedures, and the body mass index (BMI) was calculated as the weight in kilograms divided by the square of height in meters. In terms of smoking status, participants were categorized as current smokers or non-smokers. Individuals were deemed as non-drinkers if they had not consumed any alcoholic drinks in the previous year. Household income was categorized in quartiles.

The IPAQ-SF is used to estimate the overall physical activity level of an individual in metabolic equivalent (MET)-min/week by determining the duration (in minutes) and number of days (in 1 week) of engagement in three specific types of activity (walking, moderate-intensity activity, and high-intensity activity) across a comprehensive set of domains (leisure time, work-related, transport-related physical activity and domestic and gardening activity) in the past 7 days [30]. The MET is a unit used to estimate the amount of oxygen used by the body during physical activity. Subjects were classified into three PA levels (low, moderate, or high) based on the cutoff total MET-min/week in each category (IPAQ Research Committee. International physical activity questionnaire. https://sites.google.com/site/theipaq/scoring-protocol. Accessed 30 July 2016). Resistance exercise (press-up, sit-up, dumbbell, barbell, and horizontal bar exercise) was recorded according to the number of days it was performed in 1 week.

Protein intake was assessed with a 24-h dietary recall questionnaire administered by a trained dietitian. The results were generated with the food composition table developed by the National Rural Resources Development Institute (Rural Development Administration (2006) Food composition table, 7th edn. National Rural Resources Development Institute, Suwon). Inadequate protein intake was defined as protein intake under the recommended dietary reference intake for Koreans in 2015 according to the Korean Nutrition Society.

Subjects were considered to have hypertension if they had a systolic blood pressure of 140 mmHg or greater, a diastolic blood pressure of 90 mmHg or greater, or if they were being treated for hypertension. A subject was deemed to have diabetes if he or she had a fasting blood glucose level ≥ 126 mg/dL that was first detected in this survey, used an antidiabetic medication, or was previously diagnosed with diabetes by a doctor. A history of CVD was defined as a previous stroke, angina, or myocardial infarction. We used the National Cholesterol Education Program-Adult Treatment Panel III criteria to determine whether metS was present; the cutoffs for the Asia–Pacific region were employed [31]. metS was considered to be present if three or more of the following conditions were present: (i) systolic/diastolic blood pressure ≥ 130/85 mmHg or antihypertensive drug treatment, (ii) fasting serum triglycerides ≥ 150 mg/dL, (iii) low HDL-C (< 40 mg/dL in men and 50 mg/dL in women), (iv) waist circumference ≥ 90 cm in men and ≥ 80 cm in women, and/or (v) fasting serum glucose ≥ 100 mg/dL or use of antidiabetic medication. The estimated glomerular filtration rate (eGFR) was calculated based on the Modification of Diet in Renal Disease study equation [32].

### Statistical analysis

Continuous data are expressed as mean (SD), and categorical data as percentages. The first analyses explored differences in participant characteristics across the sex-specific tertiles of UA level. The chi-square test was used for categorical variables, and ANOVA was used for continuous variables.

The association between serum uric acid level and HGS was assessed through three multiple linear regression models with progressive levels of adjustment. In each of these models, the main independent variable was serum UA level (categorized into sex-specific tertiles), and the dependent variable was HGS. Model 1 was adjusted for sex and age, model 2 was additionally adjusted for BMI and income, and model 3 was additionally adjusted for current smoking, alcohol consumption, physical activity, resistance exercise, total protein intake, diabetes mellitus, hypertension, and CVD. The differential association by age group was evaluated through the inclusion of an interaction term between the UA tertiles and three pre-defined age groups (20–39, 40–59, ≥ 60) in all three models; also, the final analyses were stratified by age.

A multivariable logistic regression model was used (the enter method) to determine whether serum uric acid level was independently associated with low HGS. All statistical tests were two-tailed, and statistical significance was defined as *p* < 0.05. All statistical analyses were performed in PASW statistics 18 (SPSS Inc., Chicago, IL, USA).

## Results

### Characteristics of the subjects according to serum uric acid level stratified by age

In total, 4230 individuals (1999 male and 2231 female) were analyzed. The mean ± SD serum UA level was 5.78 ± 1.29 mg/dL in men and 4.3 ± 0.92 mg/dL in women. The sex-specific UA tertiles were ≤ 5.2 (T1), 5.3–6.2 (T2), and ≥ 6.3 (T3) mg/dL for men and ≤ 3.9 (T1), 4.0–4.6 (T2), and ≥ 4.7 (T3) mg/dL for women. The subjects were stratified by age into young (aged 20–39 years), middle-aged (aged 40–59 years), and elderly (aged ≥ 60 years) groups, which were analyzed separately. As shown in Table 1, the prevalence of obesity and metS was significantly higher in the high UA group in all age groups. Interestingly, the prevalence of diabetes mellitus was significantly lower in the high UA group in the middle-aged and elderly populations. Hypertension was more prevalent in the high UA group (T3) in the young and middle-aged groups. There was no difference in physical activity, resistance exercise, protein intake, or cardiovascular disease prevalence according to UA level. Of note, subjects in the high UA tertile had higher HGS in the young age groups in a crude comparison.

**Table 1:** Age-stratified characteristics of participants in KNHANES 2016 according to tertiles of serum uric acid levels (*N* = 4230)

Variable	Age 20–39 (*N* = 1497)					Age 40–59 (*N* = 1747)					Age ≥ 60 (*N* = 986)				
	N	T1*	T2*	T3*	*P*	N	T1*	T2*	T3*	*P*	N	T1*	T2*	T3*	*P*
		% or mean (SD)					% or mean (SD)					% or mean (SD)			
Handgrip strength	1497	32.0 (10.5)	34.2 (10.5)	35.2 (11.3)	< 0.01	1747	33.5 (10.1)	34.0 (10.3)	34.3 (10.9)	0.38	986	30.6 (9.6)	29.9 (8.9)	29.6 (9.9)	0.35
Male	650	34.9	45.9	47.4	< 0.01	796	43.7	45.7	47.6	0.17	553	63.0	53.8	48.2	< 0.01
Income					0.65					0.64					0.79
1st quartile	100	7.0	6.6	6.6		121	6.5	8.0	6.4		325	33.0	32.1	34.2	
2nd quartile	366	24.0	25.1	24.3		366	20.3	1837	23.9		292	30.6	27.8	30.5	
3rd quartile	540	36.1	38.0	34.5		544	32.9	31.9	28.4		201	21.4	22.7	16.5	
4th quartile	488	32.9	30.3	40.0		715	40.3	41.1	41.3		165	15.0	17.4	18.8	
Current smoker	372	20.5	26.4	37.6	0.04	369	19.1	20.7	23.9	0.04	125	16.0	9.7	10.9	0.03
Alcohol (ever)	1455	96.4	97.7	97.3	0.42	1617	9138	92.4	93.6	0.26	804	80.9	80.9	83.2	0.46
Physical activity (metS-h/week)	1497	1338 (2108)	1576 (2354)	1418 (1750)	0.20	1747	1335 (1974)	1278 (1565)	1284 (1569)	0.82	986	1291 (1771)	1393 (1782)	1222 (1740)	0.50
Low activity	592	44.3	35.8	39.4	0.23	765	44.0	45.7	41.7	0.71	441	43.1	43.5	48.5	0.17
Moderate activity	660	40.5	47.0	44.0		747	42.0	41.4	44.9		447	47.2	43.8	44.2	
High activity	245	15.2	17.1	16.5		235	14.0	12.9	13.4		98	9.7	12.7	7.3	
Resistance exercise (days for 1 week)	1497	1.6 (1.3)	1.7 (1.5)	1.8 (1.6)	0.05	1747	1.7 (1.5)	1.8 (1.5)	1.8 (1.5)	0.80	986	1.9 (1.7)	1.8 (1.7)	1.9 (1.7)	0.89
Protein intake (g/day)	1497	76 (45)	78 (43)	82 (50)	0.18	1747	71 (34)	73 (39)	74 (38)	0.49	986	60 (42)	61 (29)	60 (30)	0.95
Body mass index (kg/m^2^)					< 0.01					< 0.01					< 0.01
< 18.5	98	8.8	6.8	4.8		43	2.6	3.2	1.6		22	3.9	1.0	1.1	
18.5–24.9	949	71.5	68.3	53.6		1079	69.4	61.9	52.8		630	66.8	65.2	58.0	
≥ 25	444	19.7	24.9	41.6		625	28.0	34.9	45.6		334	29.3	33.8	40.9	
Hypertension	28	0.2	1.7	3.2	< 0.01	290	15.7	12.7	21.4	0.01	436	44.8	42.8	44.9	0.97
Diabetes	8	0.7	0.4	0.5	0.73	104	9.1	4.9	3.4	< 0.01	180	22.5	16.1	14.2	< 0.01
Cardiovascular disease	1	0.0	0.0	0.2	0.26	30	2.3	1.3	1.4	0.23	84	7.7	8.4	9.9	0.34
Metabolic syndrome	210	7.7	10.4	22.0	< 0.01	530	22.9	26.7	42.4	< 0.01	418	33.7	42.5	55.5	< 0.01

*T1: first tertile (≤ 5.2 mg/dL men; ≤ 3.9 mg/dL women); *T2: second tertile (5.3–6.2 mg/dL men; 4.0–4.6 mg/dL women); *T3: third tertile (≥ 6.3 mg/dL men; ≥ 4.7 mg/dL women). ANOVA was used for continuous variables. The chi-square analysis was used for categorical variables

### High uric acid level was associated with increased HGS in the elderly population after adjusting confounders

As shown in Table 1, HGS showed a significant difference according to UA level (tertile) only in young population on crude analysis. However, we observed that the UA level was positively correlated with HGS (Additional file 1: Table S1) in all age groups. When we investigated whether serum UA level was associated with HGS after adjustment for confounding factors using multivariate linear regression analysis, we observed the association of UA and HGS in the elderly population (Table 2). Model 1, which included age and sex as covariates, had similar results to the crude comparison in the young and elderly populations. However, when income (quartiles), BMI (< 18, 18–25, ≥ 25 kg/m^2^), smoking, alcohol drinking, physical activity (MET-h/week), total protein intake (g/day), hypertension, diabetes mellitus, cardiovascular disease, and resistance exercise (days/week) were taken into account (model 3), the effect of serum UA was significant only in the elderly group (T3 vs. T1: *β* coefficient [95% confidence interval (CI)] 1.017 [0.115–1.920]). We excluded subjects with rheumatoid arthritis, osteoarthritis, any cause of limitation of activity and renal insufficiency because these conditions can substantially influence the HGS or uric acid level. However, when we analyzed all the subjects with complete data set (*N* = 5431), the results were comparable (Additional file 1: Table S2). The results revealed that higher serum UA level was independently associated with higher HGS after adjustment for confounding factors in the elderly population.

**Table 2:** Multivariate linear regression analysis for handgrip strength according to tertiles of serum uric acid levels (N = 4230)

	Age 20–39 (N = 1497)				Age 40–59 (*N* = 1747)				Age ≥ 60 (*N* = 986)			
	T1*	T2*	T3*	*P*	T1*	T2*	T3*	*P*	T1*	T2*	T3*	P
	Beta coefficients (95% CI)				Beta coefficients (95% CI)				Beta coefficients (95% CI)			
Model 1	Ref	0.413 (− 0.342, 1.167)	0.994 (0.243, 1.749)	0.010	Ref	0.046 (− 0.574. 0.666)	0.188 (− 0.438, 0.814)	0.555	Ref	0.620 (− 0.197, 1.437)	1.238 (0.378, 2.098)	0.005
Model 2	Ref	0.249 (− 0.481, 0.990)	0.386 (− 0.364, 1.136)	0.312	Ref	− 0.033 (− 0.644, 0.578)	− 0.037 (− 0.661, 0.586)	0.906	Ref	0.463 (− 0.343, 1.269)	0.939 (0.084, 1.793)	0.031
Model 3	Ref	0.146 (− 0.641, 0.934)	0.093 (− 0.706, 0.892)	0.820	Ref	− 0.136 (− 0.790, 0.518)	− 0.128 (− 0.804, 0.547)	0.547	Ref	0.387 (− 0.458, 1.232)	1.017 (0.115, 1.920)	0.027

*T1: first tertile (≤ 5.2 mg/dL men; ≤ 3.9 mg/dL women); *T2: second tertile (5.3–6.2 mg/dL men; 4.0–4.6 mg/dL women); *T3: third tertile (≥ 6.3 mg/dL men; ≥ 4.7 mg/dL women)

Multivariate linear regression analysis was used

Model 1: adjusted for sex and age (per year)

Model 2: Model 1 + adjusted for income (quartile) and body mass index (< 18, 18–25, ≥ 25)

Model 3: Model 2 + adjusted for smoking, alcohol drinking, physical activity (MET-h/week), total protein intake (g/day), hypertension, diabetes, cardiovascular disease, and resistance exercise (days for 1 week)

### The association of uric acid level and HGS in the elderly population is significant in subjects with metabolic syndrome

As the prevalence of metS was significantly higher in the high UA group, the elderly population was subdivided according to the presence of metS, and the associations of serum UA level and other factors with HGS were analyzed through multivariate linear regression analysis (Table 3). Old age and female gender were negatively associated with HGS, as expected. The positive association between serum UA level (tertile) and HGS remained significant only in subjects with metS (T3 vs. T1: *β* coefficient [95% CI] = 1.64 [0.20–3.08], *p* = 0.026). In addition, more resistance exercise and higher BMI were associated with greater HGS in the aged population, regardless of the presence of metS.

**Table 3:** Multivariate linear regression analysis for handgrip strength in the older adults (age ≥ 60) according to the presence of metabolic syndrome (*N* = 986)

	No metabolic syndrome (*N* = 568)			Metabolic syndrome (*N* = 418)		
	*β*	95% CI	*P*	*β*	95% CI	*P*
Female	− 13.91	− 15.01, − 12.81	< 0.001	− 13.59	− 14.91, − 12.26	< 0.001
Age, years	− 0.40	−0.48, − 0.31	< 0.001	− 0.41	− 0.52, − 0.31	< 0.001
Uric acid						
T1*	Reference			Reference		
T2*	− 0.12	− 1.20, 0.96	0.827	1.27	− 0.16, 2.70	0.082
T3*	0.80	− 0.42, 2.03	0.196	1.64	0.20, 3.08	0.026
Income						
1st quartile	Reference			Reference		
2nd quartile	1.15	− 0.12, 2.42	0.076	1.10	− 0.38, 2.59	0.144
3rd quartile	1.15	− 0.24, 2.53	0.104	1.27	− 0.50, 3.03	0.159
4th quartile	1.71	0.25, 3.18	0.022	0.76	− 1.15, 2.67	0.435
Current smoker	0.16	− 1.30, 1.62	0.833	0.43	− 1.45, 2.32	0.651
Alcohol (ever)	0.21	− 0.80, 1.22	0.684	0.98	− 0.29, 2.24	0.131
Physical activity (metS-h/week)	0.00	0.00, 0.01	0.169	0.00	0.00, 0.00	0.930
Resistance exercise (days for 1 week)	0.31	0.03, 0.58	0.028	0.49	0.11, 0.87	0.011
Protein intake (g/day)	0.02	0.01, 0.04	0.011	0.01	− 0.00, 0.02	0.179
BMI (kg/m2)						
< 18.5	− 1.80	− 4.58, 0.99	0.205	2.47	− 3.73, 8.66	0.434
18.5–24.9	Reference			Reference		
≥ 25	2.55	1.34, 3.75	< 0.001	2.11	0.95, 3.27	< 0.001
Hypertension	0.48	− 0.54, 1.49	0.354	0.98	− 0.24, 2.21	0.116
Diabetes	− 0.48	− 2.00, 1.03	0.531	− 0.05	− 1.40, 2.32	0.948
Cardiovascular disease	−0.88	− 2.73, 0.98	0.354	−0.24	−2.17, 1.68	0.803

*T1: first tertile (≤ 5.2 mg/dL men; ≤ 3.9 mg/dL women); *T2: second tertile (5.3–6.2 mg/dL men; 4.0–4.6 mg/dL women); *T3: third tertile (≥ 6.3 mg/dL men; ≥ 4.7 mg/dL women)

Multivariate linear regression analysis was used. Sex, age, uric acid (tertile), income (quartile), current smoking, alcohol (ever), physical activity, resistance exercise, protein intake, BMI, hypertension, diabetes, and cardiovascular disease were included in the model by using the enter method

### Risk factors for low HGS in the elderly population

As serum UA level was associated with HGS only in the elderly group, and sarcopenia usually causes problems in the elderly, the risk factors for low HGS in the elderly population were determined. Among 986 subjects aged ≥ 60, 37 out of 553 (6.7%) men and 73 out of 433 (16.9%) women had low HGS (Table 4). Multivariate logistic regression analysis revealed that older age was a significant risk factor for both men (OR 1.35 [1.21–1.51]) and women (OR 1.15 [1.09–1.21]), as expected (Table 4). In addition, in male subjects, inadequate protein intake (OR 2.68 [1.06–6.81]) was a risk factor, while high serum UA level was protective (OR 0.69 [0.48–0.98]). In female subjects, diabetes mellitus was found to be a significant risk factor (OR 1.15 [1.09–1.22]); however, high serum UA level did not have a protective effect.

**Table 4:** Multivariate logistic regression analysis for low handgrip strength in the older adults (age ≥ 60) according to sex (*N* = 986)

	Men (*N* = 553), low HGS (*N* = 37)			Women (*N* = 433), low HGS (*N* = 73)		
	Odds ratio	95% CI	*P*	Odds ratio	95% CI	*P*
Age, years	1.35	1.21, 1.51	< 0.001	1.15	1.09, 1.21	< 0.001
Uric acid (mg/dL)	0.69	0.48, 0.98	0.041	0.14	0.54, 1.11	0.159
Income			0.440			0.020
1st quartile	Reference			Reference		
2nd quartile	0.58	0.18, 1.88	0.361	0.05	0.20, 0.98	0.045
3rd quartile	1.51	0.48, 4.77	0.487	0.22	0.06, 0.78	0.019
4th quartile	0.33	0.04, 3.17	0.340	0.31	0.09, 0.99	0.050
Current smoker	0.84	0.28, 2.55	0.754	0.90	0.14, 2.19	0.916
Alcohol (ever)	1.50	0.59, 3.86	0.398	0.71	0.36, 1.39	0.320
Physical activity (metS-h/week)			0.318			0.930
Low	Reference					
Moderate	0.53	0.22, 1.32	0.172	0.95	0.49, 1.86	0.883
High	0.37	0.04, 3.32	0.377	0.76	0.36, 1.39	0.709
Resistance exercise (≥ 2 days/week)	0.37	0.09, 1.48	0.160	0.53	0.16, 1.71	0.287
Inadequate protein intake	2.68	1.06, 6.81	0.038	1.15	0.60, 2.22	0.670
BMI (kg/m2)			0.800			0.157
< 18.5	2.09	0.17, 25.5	0.564	2.51	0.37, 17.09	0.347
18.5–24.9	Reference			Reference		
≥ 25	1.70	0.11, 26.78	0.708	1.32	0.19, 9.40	0.783
Hypertension	0.97	0.38, 2.48	0.956	0.63	0.31, 1.28	0.202
Diabetes	0.54	0.17, 1.78	0.543	1.15	1.09, 1.22	0.044
Cardiovascular disease	0.48	0.12, 1.89	0.482	0.55	0.14, 2.22	0.547
Metabolic syndrome	1.45	0.48, 4.33	0.510	1.46	0.72, 2.99	0.298

Multivariate logistic regression analysis was used. Sex, age, uric acid, income (quartile), current smoking, alcohol (ever), physical activity, resistance exercise, protein intake, BMI, hypertension, diabetes, cardiovascular disease, and metabolic syndrome were included in the model by using enter method

## Discussion

In the current study, we verified that a high serum UA level is independently associated with increased HGS in the Korean elderly population. The protective effect of UA was significant in subjects with metS. In subjects aged 20–59 years, the association of UA with HGS was not observed after adjustment for confounding factors.

HGS measured with a hand-held dynamometer is known to properly represent overall muscle strength [26]. HGS depends not only on age, sex, and BMI, but also on socioeconomic status, physical activity, and cognitive function. As HGS declines with aging, it may represent the overall well-being of the elderly [33]. Indeed, HGS is associated with mortality in the elderly population [34]. Therefore, the maintenance of HGS is important, and the identification of factors affecting HGS is valuable, especially in the elderly population.

Our findings are consistent with the results of previous studies conducted in the elderly population. Macchi et al. [19] demonstrated that high baseline UA level was associated with better HGS at 3 years of follow-up in the elderly. A Japanese study conducted with 602 men aged 72 ± 7 years and 847 women aged 71 ± 6 years indicated that, after adjustment for confounding factors, high serum UA level was independently associated with higher HGS only in women [21]. More recently, Molino-Lova et al. [22] also demonstrated that, in a very old population (mean age 92.8 ± 3.1 years) consisting of 73 men and 166 women, higher serum UA levels correlated with better HGS.

As mentioned, studies on the relationship between UA level and muscle strength in the younger population are few; however, two recent studies included younger and elderly subjects. Huang et al. [24] reported that hyperuricemia was associated with poor muscle strength. Japanese men over 30 years of age were enrolled in the study, and 630 subjects were analyzed; however, there was no subgroup analysis stratified by age. The other study was conducted among 3595 individuals who participated in the NHANES study [25]. We used similar statistical analysis strategy with this study including the usage of sex-specific tertiles of uric acid as the main independent variable. Consistent with our results, the authors demonstrated that high serum UA level was associated with better HGS in subjects aged ≥ 60 years. Interestingly, they found a detrimental effect of UA in the young population, whereas we found no significant relationship between UA level and HGS in the young population of our study. This discrepancy seems to be partly due to the different serum UA levels of the study subjects; the mean UA levels in the young groups of both sexes were lower in our study (5.78 ± 1.29 vs. 6.0 ± 1.2 mg/dL in men, 4.3 ± 0.92 vs. 4.8 ± 1.3 mg/dL in women). Considering the results of the three studies, including ours, age seems to modify the effect of UA on HGS.

In the current study, the serum UA level of the majority of subjects was within the normal range, and only a small number of individuals had hyperuricemia, even in the T3 group.

Interestingly, we found that high serum UA level had a protective effect on HGS in elderly subjects with metS. The prevalence of metS increased with age as well as with serum UA level, and 55.5% of the elderly T3 group had metS, compared with 22.0% and 42.4% in the young and middle-aged T3 groups, respectively. On the contrary, an increased prevalence of metS in subjects with sarcopenia has been reported [35]. Several potential mechanisms underlying the association of metS with sarcopenia have been proposed. One is that reduced total muscle mass increases insulin resistance. The selective loss of muscle fibers that produce myokines that act against pathologic adipokines during aging also seems to contribute to the association between metS and sarcopenia. Although the exact explanation for the protective effect of UA in metS may require further research, the finding is novel and intriguing.

Low serum UA level was a risk factor for low HGS only in the male elderly population. These results were the opposite of those of Kawamoto et al. [21], who found that the serum UA level was independently associated with HGS only in women. This gender difference in the study is less conclusive because of the small number of low HGS subjects among men (110 out of 602). The study might be underpowered due to the small subgroup number and fail to detect a small association. However, we can assume possible reasons for the discrepancy of the results in the male population between the two studies. One plausible explanation is that the combined effects of different hormones, alcohol consumption rate, different mean UA level, and smoking habits result in this sex difference.

The major limitation of the current study was its cross-sectional design, which hampered our ability to determine causality. However, this study also had strengths in that it was conducted with a large and unbiased population with enough information for the adjustment of potential confounding factors. Also, as the seventh KNHANES did not include a tool to evaluate muscle mass such as dual-energy x-ray absorptiometry, we could not determine the muscle mass component, which is critical for determining sarcopenia.

## Conclusions

This population-based cross-sectional survey in Korea revealed that high serum UA level is associated with increased HGS in the aged population. UA seems to enhance muscle strength, especially in elderly individuals with metS.

## Additional file

Additional file 1: **Table S1.** Correlation between serum uric acid and handgrip strength stratified by age group. Table S2. Multivariate linear regression analysis for handgrip strength according to the group of age (*N* = 5431). (DOCX 22 kb)

## Abbreviations

BMI: Body mass index
CI: Confidence interval
DAMP: Danger-associated molecular pattern
HGS: Hand grip strength
KNHANES: Korea National Health and Nutrition Examination Survey
MET: Metabolic equivalent
metS: Metabolic syndrome
OR: Odds ratio
UA: Uric acid
