# Supplementary material for: High serum uric acid level is associated with greater handgrip strength in the aged population
Source: Arthritis Res Ther. 2019 Mar 12;21:73. doi: 10.1186/s13075-019-1858-2 (PMC6417193; doi:10.1186/s13075-019-1858-2)
Supplement: Supplementary file 1 — Table S1. Correlation between serum uric acid and handgrip strength stratified by age group. Table S2. Multivariate linear regression analysis for handgrip strength according to the group of age (N = 5431). (DOCX 22 kb) [file 13075_2019_1858_MOESM1_ESM.docx]

**Table S1** Correlation between serum uric acid and handgrip strength stratified by age group.

| Age group | handgrip strength (kg) | |
| --- | --- | --- |
|  | r | *p*-value |
| 20-39, serum uric acid | 0.571 | <0.001 |
| 40-59, serum uric acid | 0.475 | <0.001 |
| ≥ 60, serum uric acid | 0.372 | <0.001 |

Pearson’s correlation analysis was used.

**Table S2** Mutivariate linear regression analysis for handgrip strength according to the group of age (N=5431).

|  | Age 20-39 (N=1597) | | |  | Age 40-59 (N=2066) | | |  | Age ≥ 60(N=1768) | | |
| --- | --- | --- | --- | --- | --- | --- | --- | --- | --- | --- | --- |
|  | β | 95% CI | P |  | β | 95% CI | P |  | β | 95% CI | P |
| Female | -15.67 | -16.53, -14.85 | <0.001 |  | -16.72 | -17.44, -15.99 | <0.001 |  | -13.34 | -14.12, -12.55 | <0.001 |
| Age, year | 0.16 | 0.10, 0.21 | <0.001 |  | -0.13 | -0.18, -0.08 | <0.001 |  | -0.37 | -0.43, -0.32 | <0.001 |
| Uric acid |  |  |  |  |  |  |  |  |  |  |  |
| T1* | Reference | | |  | Reference | | |  | Reference | | |
| T2* | -0.09 | -0.94, 0.76 | 0.839 |  | -0.74 | -0.75, 0.61 | 0.831 |  | 0.34 | -0.41, 1.10 | 0.375 |
| T3* | 0.04 | -0.83, 0.91 | 0.935 |  | 0.38 | -0.67, 0.74 | 0.917 |  | 0.85 | 0.05, 1.65 | 0.036 |
| Income |  |  |  |  |  |  |  |  |  |  |  |
| 1st Quartile | Reference | | |  | Reference | | |  | Reference | | |
| 2nd Quartile | 0.71 | -0.47, 2.33 | 0.192 |  | 0.62 | -0.54, 1.78 | 0.295 |  | 0.86 | 0.07, 1.65 | 0.033 |
| 3rd Quartile | 1.05 | -0.29, 2.39 | 0.124 |  | 1.80 | 0.68, 2.93 | 0.002 |  | 0.90 | -0.02, 1.81 | 0.054 |
| 4th Quartile | 1.14 | -0.20, 2.49 | 0.096 |  | 1.81 | 0.78, 2.94 | 0.001 |  | 1.01 | 0.05, 1.65 | 0.040 |
| Current smoker | 1.48 | 0.60, 2.36 | 0.001 |  | 0.00 | -0.81, 0.80 | 0.993 |  | 0.51 | -0.57, 1.58 | 0.355 |
| Alcohol (ever) | 4.46 | 2.48, 6.44 | <0.001 |  | 0.50 | -0.52, 1.52 | 0.333 |  | 0.29 | -0.50, 1.08 | 0.468 |
| Physical activity  (METS-h/week) | 0.00 | 0.00, 0.00 | 0.771 |  | 0.00 | 0.00, 0.00 | 0.981 |  | 0.00 | 0.00, 0.00 | 0.004 |
| Resistance exercise  (days for 1 week) | 0.63 | 0.34, 0.87 | <0.001 |  | 0.23 | 0.05, 0.42 | 0.014 |  | 0.40 | 0.22, 0.59 | <0.001 |
| Protein intake  (g/day) | 0.01 | 0.00, 0.02 | 0.044 |  | 0.00 | 0.00, 0.12 | 0.249 |  | 0.02 | 0.01, 0.03 | 0.002 |
| BMI (kg/m2) |  |  |  |  |  |  |  |  |  |  |  |
| <18.5 | -2.53 | -3.87, -1.19 | <0.001 |  | -1.94 | -3.68, -0.19 | 0.030 |  | -2.78 | -4.85, -0.71 | 0.009 |
| 18.5-24.9 | Reference | | |  | Reference | | |  | Reference | | |
| ≥25 | 2.02 | 1.25, 2.79 | <0.001 |  | 1.42 | 0.80, 2.04 | <0.001 |  | 1.45 | 0.81, 2.10 | <0.001 |
| Hypertension | 1.57 | 0.23, 2.92 | 0.022 |  | 0.68 | 0.00, 1.37 | 0.052 |  | 0.78 | 0.15, 1.42 | 0.016 |
| Diabetes | -1.16 | -3.62, 1.29 | 0.354 |  | -0.71 | -1.69, 0.27 | 0.158 |  | -0.21 | -0.93, 0.51 | 0.57 |
| Cardiovascular disease# | - | - | - |  | -1.79 | -3.85, 0.27 | 0.089 |  | -0.77 | -1.77, 0.23 | 0.132 |
| eGFR < 60 (mL/min/1.73m²) | 2.62 | 0.61, 4.63 | 0.011 |  | 0.71 | -0.29, 1.71 | 0.165 |  | 0.40 | -0.42, 1.21 | 0.339 |

*T1: First tertile (≤5.1 mg/dl men; ≤3.8 mg/dl women); *T2: Second tertile (5.2-6.2 mg/dl men; 3.9-4.6 mg/dl women); *T3: Third tertile (≥6.3 mg/dl men; ≥4.7 mg/dl women)

^#^Cardiovascular disease was observed in only 1 subject among age group 1 (age 20-39). All variables were included in the model using enter method.
